# Characteristics of Globus Pallidus Internus Local Field Potentials in Hyperkinetic Disease

**DOI:** 10.3389/fneur.2018.00934

**Published:** 2018-11-05

**Authors:** Guanyu Zhu, Xinyi Geng, Zheng Tan, Yingchuan Chen, Ruili Zhang, Xiu Wang, Tipu Aziz, Shouyan Wang, Jianguo Zhang

**Affiliations:** ^1^Department of Neurosurgery, Beijing Tiantan Hospital, Capital Medical University, Beijing, China; ^2^Institute of Science and Technology for Brain-Inspired Intelligence, Fudan University, Shanghai, China; ^3^Department of Psychology, University of Chinese Academy of Sciences (UCAS), Beijing, China; ^4^Medical Sciences Division, Nuffield Department of Surgical Sciences, University of Oxford, Oxford, United Kingdom; ^5^Beijing Key Laboratory of Neurostimulation, Beijing, China; ^6^Department of Functional Neurosurgery, Beijing Neurosurgical Institute, Beijing Tiantan Hospital, Capital Medical University, Beijing, China

**Keywords:** Huntington disease (HD), dystonia, globus pallidus internus (GPi), local field potential (LFP), deep brain brain stimulation

## Abstract

**Background:** Dystonia and Huntington's disease (HD) are both hyperkinetic movement disorders but exhibit distinct clinical characteristics. Aberrant output from the globus pallidus internus (GPi) is involved in the pathophysiology of both HD and dystonia, and deep brain stimulation (DBS) of the GPi shows good clinical efficacy in both disorders. The electrode externalized period provides an opportunity to record local field potentials (LFPs) from the GPi to examine if activity patterns differ between hyperkinetic disorders and are associated with specific clinical characteristics.

**Methods:** LFPs were recorded from 7 chorea-dominant HD and nine cervical dystonia patients. Differences in oscillatory activities were compared by power spectrum and Lempel-Ziv complexity (LZC). The discrepancy band power ratio was used to control for the influence of absolute power differences between groups. We further identified discrepant frequency bands and frequency band ratios for each subject and examined the correlations with clinical scores.

**Results:** Dystonia patients exhibited greater low frequency power (6–14 Hz) while HD patients demonstrated greater high-beta and low-gamma power (26–43 Hz) (*p* < 0.0298, corrected). United Huntington Disease Rating Scale chorea sub-score was positively correlated with 26–43 Hz frequency band power and negatively correlated with the 6–14 Hz/26–43 Hz band power ratio.

**Conclusion:** Dystonia and HD are characterized by distinct oscillatory activity patterns, which may relate to distinct clinical characteristics. Specifically, chorea may be related to elevated high-beta and low-gamma band power, while dystonia may be related to elevated low frequency band power. These LFPs may be useful biomarkers for adaptive DBS to treat hyperkinetic diseases.

## Introduction

Many movement disorders involve basal ganglia dysfunction, including Parkinson‘s disease (PD), dystonia, essential tremor, Huntington's disease (HD), and Tourette syndrome (TS). Furthermore, symptoms of these diseases can be alleviated by deep brain stimulation (DBS) (1–5). The introduction of DBS and the electrodes externalized period provides an opportunity to learn more about the pathology of the disorders (6).

Beta oscillations in the sub-thalamic nucleus (STN) are correlated with the rigidity syndrome of PD. Alpha oscillations in the pedunculopontine nucleus (PPN) correlate with gait performance in PD (7). In dystonia patients, a lower frequency band is identified (8, 9). In cervical dystonia patients, the theta oscillation is strongly correlated to dystonic symptoms (10). Dystonic involuntary muscle spasms are specifically related to the elevated theta, alpha, and low-frequency beta (low-beta) power (3–18 Hz) (11), and a study of 13 dystonia patients found that 4–10 Hz had the greatest band power.

A limitation of such studies is the absence of a true control group (i.e., neurologically healthy individuals) as no such group would have implanted DBS electrodes, so many studies have instead compared LFPs among patients with different movement disorders. One study comparing LFPs between dystonia and PD patients found that dystonia patients had lower 11–30 Hz power and greater 4–10 Hz power than PD patients (12), and another found that the peak frequency of LFP oscillations was lower in dystonia than PD (13). Our previous work also demonstrated that dystonia patients off medication had lower beta power but greater low frequency and high-gamma power than PD patients (14).

Combined the above evidence, some disease related abnormal LFP overlap with each other. Some researchers speculate the abnormal LFP might not relate to a specific disease but relate to a specific syndrome (14, 15). We also agree with this opinion. As many previous studies report the characteristic of dystonia, we chose another hyperkinetic disorders HD which have some same and different syndromes to add some evidence on this hypothesis.

In general, HD patients demonstrate a good therapeutic response to DBS (16–18) and the GPi is a frequent target for DBS-therapy in HD (19). However, GPi-LFPs have not been examined extensively in HD. One such study comparing the oscillation patterns between a single HD patient and a group of 14 PD patients found that the 3–35 Hz oscillations are not a prominent feature of HD and provided support for the theory that beta activity is primarily “antikinetic” (20). Another study investigated the LFP in a 65-year-old female HD patient and found that low-gamma activity (35–45 Hz) was elevated and reflected pathological exaggeration of the motor drive. Alpha/theta (4–12 Hz) oscillation is also prominent in the GPi among HD patients, suggesting that elevated alpha/theta oscillation may be a common feature of disorders with involuntary movement (21). The mentioned abnormal LFPs in HD also have overlap with the dystonia. As dystonia and HD are both hyperkinetic disorders, but with distinct clinical features, we hypothesized the LFPs of HD and dystonia may have different oscillation patterns that explain unique clinical characteristics.

## Materials and methods

### Subjects and surgery

The study was performed according to the Declaration of Helsinki and approved by the local ethics committees of the two participating centers. Informed written consent was provided by all patients. Seven patients with chorea-dominant HD [age 46.8 (SD 12.9) years; disease duration: 6.8 (SD 2.1) years; five males and two females] participated in this study. All patients stopped the medication 1 week before DBS surgery. A group of nine subjects with dystonia [age 51.3 (SD 13.5) years; disease duration: 11.2 (SD 2.9) years; eight males and one female] were also recruited, all with primary cervical dystonia. The United Huntington Disease Rating Scale chorea sub-score (UHDRS-c) was used to assess chorea syndrome severity in HD patients, while dystonic syndrome was quantified using the Burke-Fahn-Marsden dystonia rating scale (BFMDRS) or Toronto Western Spasmodic Torticollis Scale (TWSTRS). Further details on patient demographics and clinical characteristics are summarized in Table 1.

**Table 1:** Clinical summary.

**Case**	**Age/Sex**	**Dominated symptoms**	**Duration of disease (years)**	**Pre-operative scales (HD: UHDRS-C UHDRS-D; Dystonia: BFMDRS/TWSTRS)**	**Channel selection**
H1	31/F	Chorea	7	C:24 D:0	L12, R01
H2	42/M	Chorea	6	C:26 D:0	L12, R12
H3	35/F	Chorea Dysarthria	5	C:14 D:2	L01, R01
H4	42/F	Chorea	4	C:16 D:2	L01, R12
H5	63/M	Chorea Dysarthria	7	C:20 D:0	L12, R12
H6	51/M	Chorea	10	C:19 D:0	L12, R23
H7	64/M	Chorea	9	C:23 D:3	L23, R12
D1	59/M	Primary Cervical	7	TWSTRS:64	L01, R12
D2	46/F	Primary Cervical	10	BFMDRS:77	L01, R01
D3	33/M	Primary Cervical	12	BFMDRS:85	L12, R12
D4	36/M	Primary Cervical	14	TWSTRS:40	L01, R12
D5	79/M	Primary Cervical	14	TWSTRS:57	L12, R01
D6	49/M	Primary Cervical	12	BFMDRS:63	L12, R01
D7	57/M	Primary Cervical	10	TWSTRS:70	L01, R12
D8	50/M	Primary Cervical	7	TWSTRS:80	L12, R23
D9	53/M	Primary Cervical	15	TWSTRS:57	L12, R23

*UHDRS-C, UHDRS chorea subscores; UHDRS-D, UHDRS dystonia subscores*.

For the HD group, six patients underwent bilateral GPi implantation and one patient (H7) underwent bilateral implantation in both STN and GPi (14 GPi sides). All dystonia patients also received bilateral GPi implantation (18 sides). The electrodes were aimed at the posterior-lateral GPi in both groups, which is reported to form part of the motor circuit. The targets were calculated and determined using the SurgiPlan system (ELEKTA, Stockholm, Sweden). The DBS electrodes were Medtronic 3387 (Medtronic, Minneapolis, MN, USA) and PINS L302 (PINS, Beijing, China) with four platinum-iridium cylindrical surface contacts. Each contact was 1.27 mm in diameter and 1.5 mm in length, and contacts were spaced 1.5 mm from one another. The most caudal contact was termed contact 0 and the most rostral contact 3.

### Recording

All recordings were conducted with the patient in the sitting position. Patients were not instructed to suppress any involuntary movement but were asked to avoid voluntary movement during recording. Three channels were simultaneously recorded from the adjacent pairs of four contacts (0–1, 1–2, and 2–3) with a common electrode placed on the surface of the mastoid. The locality of the GPi-LFPs was reflected by the graded changes in both amplitude and frequency of the oscillatory activity recorded via different pairs of contacts. Artifacts were carefully identified and marked on the recordings for exclusion from analysis. The signals were amplified, bandpass filtered over 1–1,000 Hz using a Digitimer Amplifier (Model D360, Digitimer Ltd., Hertfordshire, UK) and recorded at a sampling frequency of 2 kHz using a CED 1401 data acquisition interface (Cambridge Electronic Design, Cambridge, UK). All data were displayed on line in Spike2 (Cambridge Electronic Design) and then exported to spread sheet format for analysis.

### Signal processing

All signals were down-sampled to 500 Hz for further analysis. A custom script developed in MATLAB (MathWorks Inc., Natick, MA, USA) was used for signal processing, data analysis, and statistical analysis. A continuous 20-s artifact-free signal was selected from one bipolar channel on each side for analysis (all channels selected are shown in Table 1).

### Spectral analysis

Signals were bandpass filtered between 3–45 Hz and then z-transformed to cancel the variance between subjects. Welch's method (22) was used to calculate the power spectral density with a 1-s sliding window and 0.5 s overlap. The normalized power spectra were compared every 0.5 Hz over 3–45 Hz between the dystonia and HD groups using unpaired *t*-tests. For multiple comparisons across frequencies, we chose false discovery rate (FDR) correction for significance and required significance in three or more consecutive bins. The mean total power values in discrepant frequency bands were compared between the dystonia and HD groups by *t*-test.

### Lempel-ziv complexity (LZC) measurement

The LZC measurement is related to the number of distinct substrings or patterns within the sequence and the rate of their occurrence along the sequence, which is a non-parametric measure of Kolmogorov complexity for finite sequences (23). More complex signals will contain a greater variety of distinct patterns than simpler (more regular) signals. Non-linear LFP analysis using LZC measurement is an important supplement to results obtained by spectral analyses (24, 25). The pre-processed signals were band-pass filtered over 6–14, 15–25, and 26–43 Hz (the bands identified by spectral analysis as discrepant between groups). The individual neural oscillations of band LFPs were converted into binary sequences using the median value of the oscillation amplitude as the threshold. The LZC measurement was applied to every 1-s epoch and averaged. Then the number of different substrings or patterns in the binary sequence was computed (23). *T*-tests were used to compare the complexity difference between frequency bands.

### Power ratio analysis and the correlation with clinical assessment

The oscillatory balance between two oscillations can be represented by ratio of power accumulated over the two frequencies (26) and such measurement has been showed to be related to brain functions. In this case, the power ratio of the discrepant frequencies, that is 6–14 and 26–43 Hz were calculated and analyzed. The power ratios between the discrepancy frequencies were then used to calculate the receiver operating characteristic (ROC) and find the optimum value to differentiate HD from dystonia. Pearson's correlation coefficients (Rs) were calculated to assess the correlation strengths of the clinical assessment scores with discrepant frequency band powers and band power ratios. The UHDRS-c was used as a measure of syndrome severity for HD (*n* = 7) and TWSTRS for dystonia (*n* = 6). Three dystonia patients were excluded because the clinical assessment was conducted using BFMDRS. The *p* < 0.05 was considered statistically significant. All data were plotted using MATLAB and GraphPad Prism version 6.00 (GraphPad Software, La Jolla, CA, USA).

## Results

### GPi targeting and raw data

**Figure 1:**
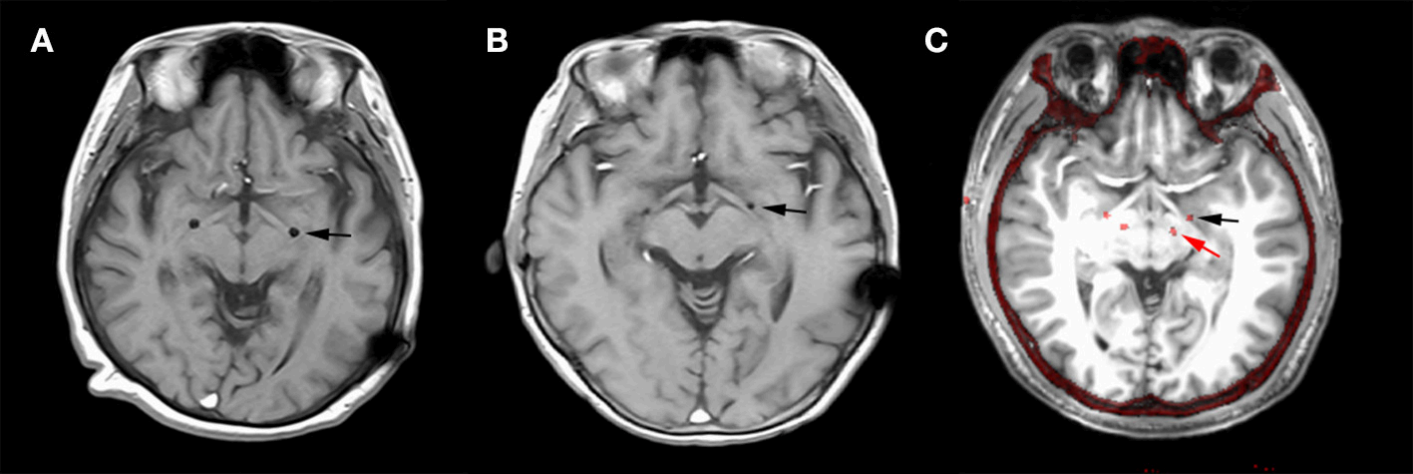
Deep brain stimulation (DBS) lead locations. Representative examples of lead locations in the left (L)-GPi of patients H2 and H6 and in both L-STN and L-GPi of patient H7 derived from post-operative MR and merged post-operative CT with pre-operative MRI. The black asterisk represents the location of GPi and the red asterisk represents the location of STN. **(A)** Post-operative MR showing the lead location in GPi of patient H1 on the horizontal plane. **(B)** Post-operative MR showing the lead location in GPi of patient H2 on the horizontal plane. **(C)** Merged image showing the lead locations in GPi and STN of patient H7 on the horizontal plane.

**Figure 2:**
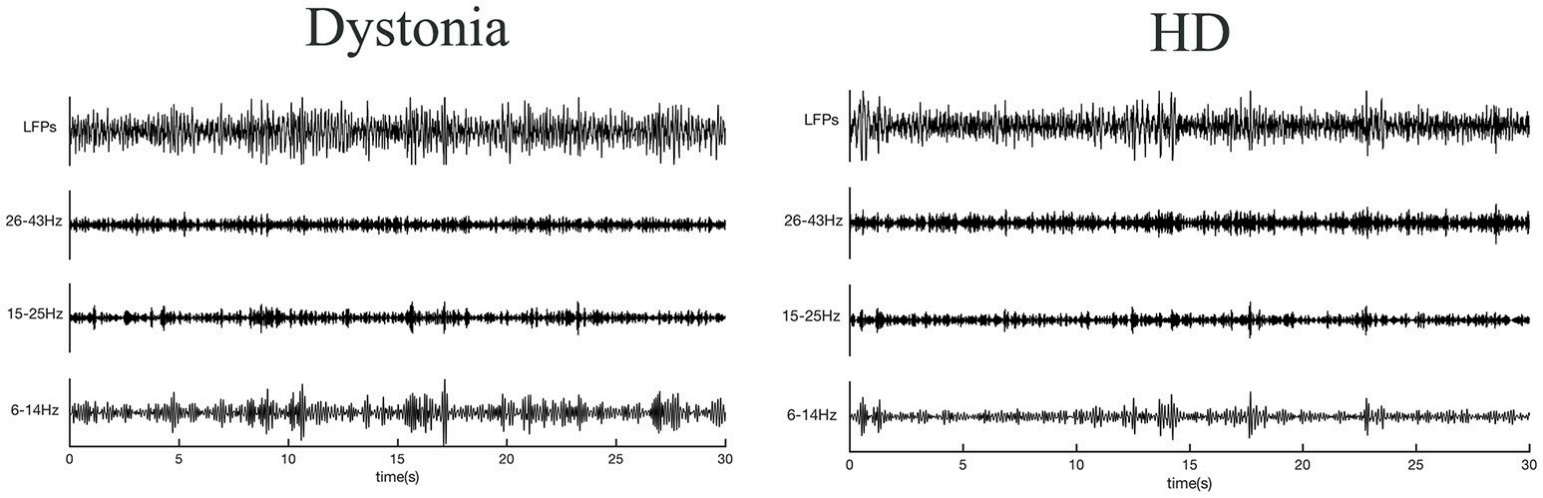
Raw local field potentials (LFPs) from dystonia and HD patients. Signals predominantly within 6–14, 15–25, and 26–43 Hz bands are presented.

The post-operative MRI of patients H1 and H2 with electrodes implanted in GPi and the merged image of H7 with electrodes implanted in both GPi and STN are shown in Figure 1. All targets were toward the posterior-lateral GPi. The target coordinates in left and right hemispheres were as follows: Left Lateral: 20.1 ± 1.27 mm, Left Anterior: 5.9 ± 1.43, Left Inferior: 4.5 ± 0.98; Right Lateral: 19.5 ± 1.50 mm, Right Anterior: 5.9 ± 1.32, Right Inferior: 4.5 ± 1.08. The mid-commissural point (MCP) was taken as the origin and coordinates for the GPi were taken from the Atlas of Stereotaxy of the Chinese human brain based on MRI scans. The raw data and band filter data are shown in Figure 2.

### Different spectral characteristics in HD and dystonia

**Figure 3:**
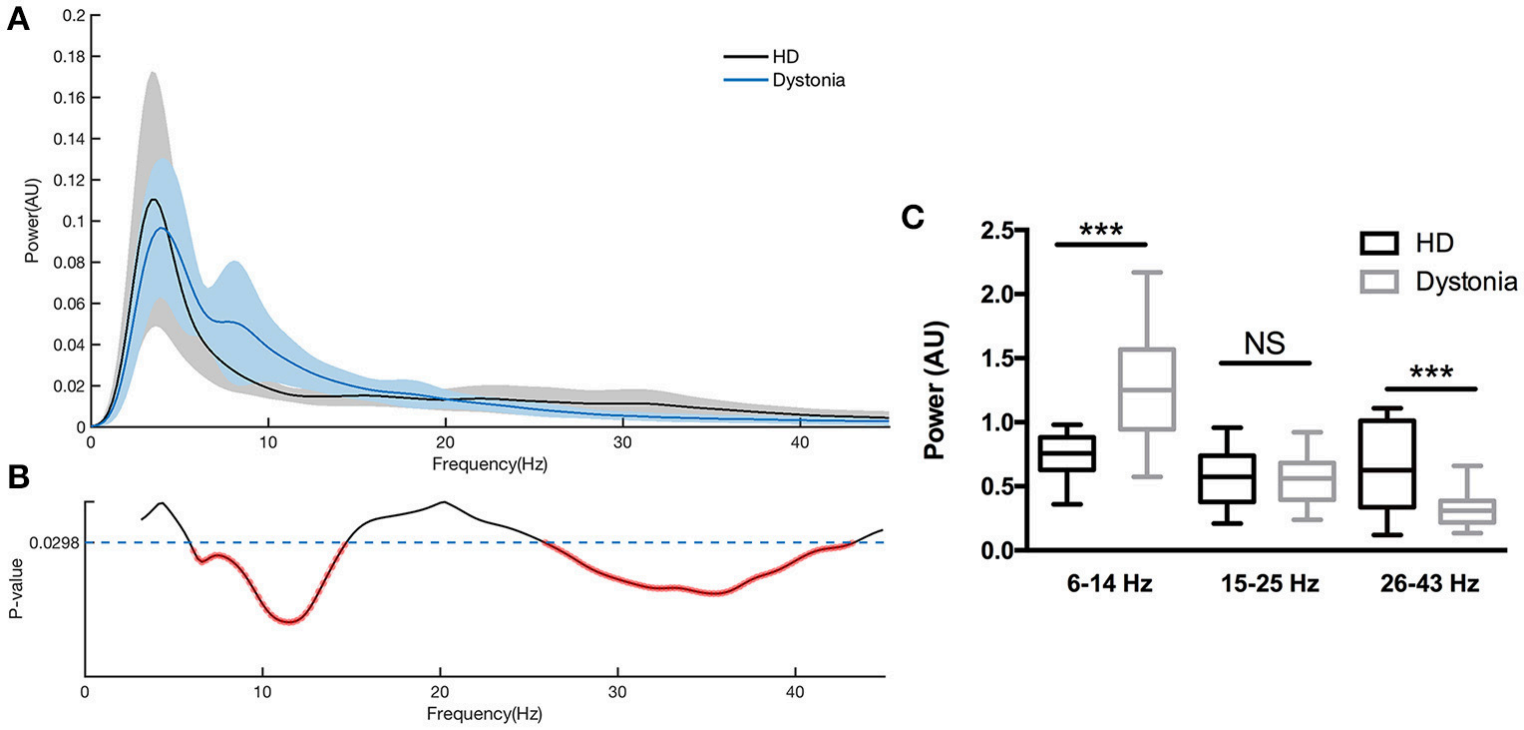
Power spectra over 3–45 Hz across subjects with dystonia or HD. Groups were compared by two-sample, unpaired *t*-tests. LFPs were z-transformed before spectral analysis. Spectral resolution is 0.5 Hz. **(A)** The blue line and shadows represents the average (± standard error) power of the dystonia group and the gray line represent the average (± standard error) power of the HD group. **(B)** Statistical comparisons of band powers between dystonia and HD groups. Significant differences were found for 6–14 and 25–43 Hz bands. **(C)** The mean total power values at 6–14, 15–25, and 26–43 Hz for each group are shown in the histogram. Significant group differences were found for the 6–14 and 26–43 Hz bands (*p* < 0.0298, corrected *p*-value). No significant group difference was found for the 15–25 Hz band. ***p < 0.05.

The normalized power spectral densities of 14 sides from 7 HD patients and 18 sides from 9 dystonia patients are shown individually and averaged within groups in Figure 3. The HD group exhibited elevated spectral power in the high-beta and low-gamma bands, while the dystonia group showed elevated low frequency power (Figure 3A). Contiguous significant differences in power were found over 6–13 Hz (two-sample unpaired *t*-tests, *p* < 0.0298 in each of 3 contiguous bins respectively) and over 28–45 Hz (*p* < 0.0298 in each of 3 contiguous bins) between HD and dystonia groups (Figure 3B). The individual total band power values at 6–14, 15–25, and 26–43 Hz were compared between groups and significant differences were found over 6–14 and 26–43 Hz (two-sample unpaired *t*-tests, *p* < 0.05; Figure 3C).

### Dynamic analysis in HD and dystonia

**Figure 4:**
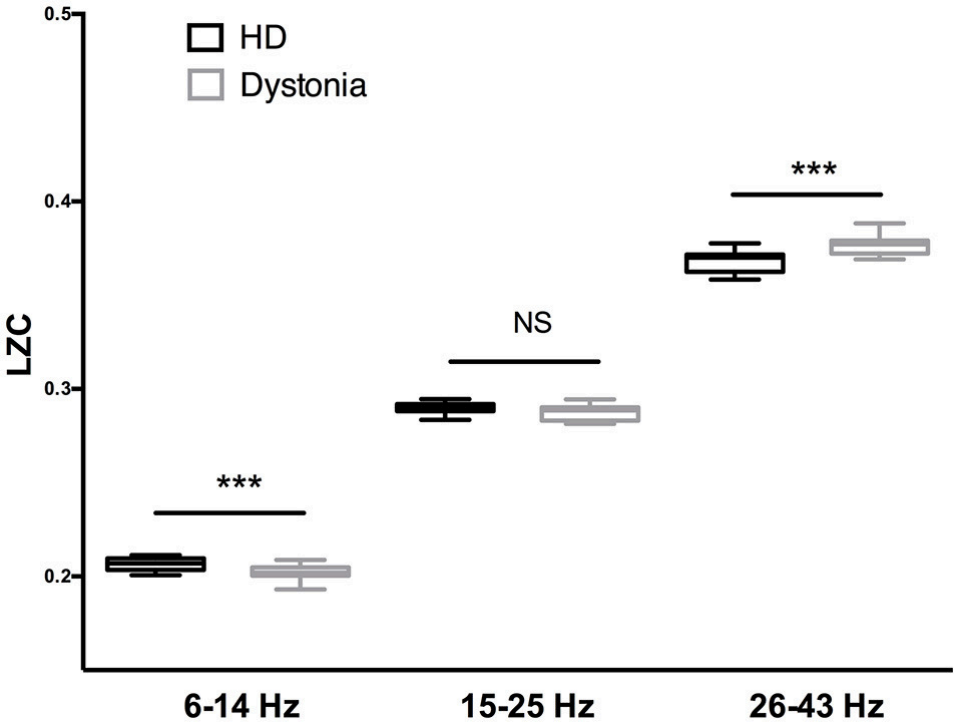
Complexity analysis of HD and dystonia LFPs centered on the peak frequency in 6–14, 15–25, and 26–43 Hz bands. Significant differences in LZC were found between HD and dystonia groups at 6–14, 15–25, and 26–43 Hz [*F*_(5, 90)_ = 28.18, *p* < 0.0001, one-way ANOVA]. The LZC of the 6–13 Hz band was significantly lower in the dystonia group than the HD group (*t*-tests, *p* ≤ 0.001). The LZC values in the high-beta and low-gamma bands were significantly higher in the dystonia group than the HD group (*t*-tests, ***p < 0.001).

The LZC over the 6–13 Hz band was significantly lower in the dystonia group than the HD group (*t*-tests, *p* < 0.001). In other words, the oscillations within this band were less complex or more regular in the dystonia group. Conversely, the LZC measures in the high-beta and low-gamma band were significantly higher in the dystonia group than the HD group (*t*-tests, *p* < 0.001). No significant difference in LZC was found in the 14–27 Hz band (Figure 4).

### Power ratio and clinical assessment correlation

**Figure 5:**
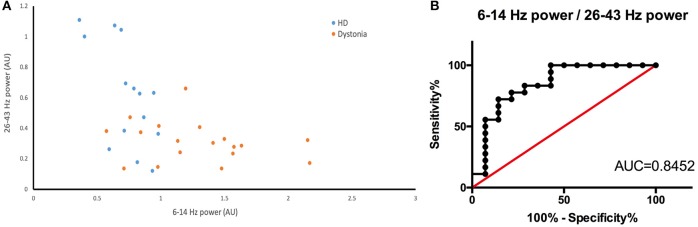
**(A)** Scatter plot of 26–43 Hz power (X-axis) vs. 6–14 Hz power (Y-axis) for dystonia patients (yellow dots) and HD patients (blue dots). There is a clear boundary between the two groups. **(B)** The ROC constructed from the 6–14 Hz/26–43 Hz of HD and dystonia patients (AUC is 0.8452).

**Figure 6:**
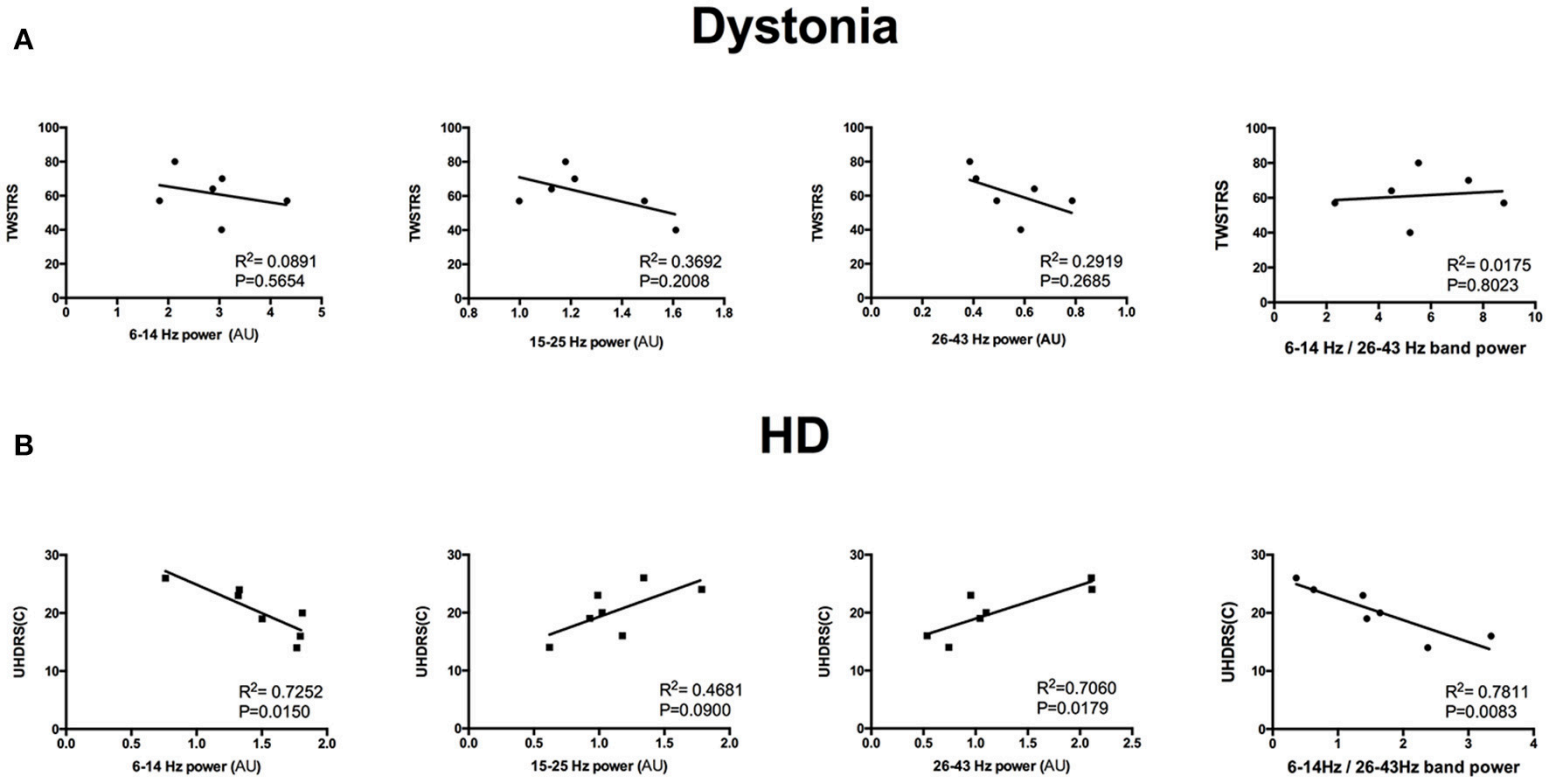
**(A)** Dystonia severity (TWSTRS) was not correlated with total bilateral spectral power in the 6–14, 15–25, and 26–43 Hz bands or with the 6–14 Hz/26–43 Hz ratio (*n* = 6; all *P* < 0.05). **(B)** HD chorea severity (UHDRS-c) was positively correlated with 26–43 Hz frequency band power and negatively correlated with the 6–14 Hz/26–43 Hz band power ratio (*n* = 7; all *P* < 0.05).

The HD group and the dystonia group demonstrated obvious differences in 6–14 and 26–43 Hz power bands (Figure 5A) and these differences could be used to distinguish patient groups. For instance, the groups were well differentiated by the 6–14 to 26–43 Hz power ratio, with greatest likelihood of 7.778 at a ratio >4.666 (with area under the ROC curve of 0.8452; Figure 5B). The TWSTRS score showed no significant correlations with the total bilateral power at 6–14, 15–25, and 26–43 Hz, or with the 6–14 Hz/26–43 Hz ratio (*n* = 6; 6–14 Hz: *R*^2^ = 0.0891, *P* = 0.5654; 15–25 Hz: *R*^2^ = 0.3692, *P* = 0.2008; 26–43 Hz: *R*^2^ = 0.2919, *P* = 0.2685; 6–14 Hz/26–43 Hz: *R*^2^ = 0.0175, *P* = 0.8023; Figure 6A). In contrast, the UHDRS-c was positively correlated with 26–43 Hz frequency band power and negatively correlated with the 6–14 Hz/26–43 Hz band power ratio (*n* = 7; 6–14 Hz: *R*^2^ = 0.7252, *P* = 0.0150; 15–25 Hz: *R*^2^ = 0.4681, *P* = 0.0900; 26–43 Hz: *R*^2^ = 0.7060, *P* = 0.0179; 6–14 Hz/26–43 Hz: *R*^2^ = 0.7811, *P* = 0.0083; Figure 6B).

## Discussion

Few studies have investigated GPi-LFP anomalies in HD. By comparing the GPi-LFP patterns between HD patients and dystonia patients, we demonstrate significant differences in spectral power in the 6–14 and 26–43 Hz frequency bands that may explain the predominant movement deficit in each group. These LFP recordings may provide clues to the pathological changes in the GPi contributing to HD and dystonia symptoms.

### Clinical characteristics of the study groups

Dystonia and HD are both hyperkinetic disorders but with distinct clinical presentation. Dystonia is characterized by sustained muscle contractions, causing abnormal postures (tonic component) or repetitive movements (phasic component). Typical clinical features of dystonic contractions are marked directionality, long duration, and simultaneous involvement of agonist and antagonist muscles (27). In contrast, chorea in HD is characterized by rapid, purposeless, non-stereotyped movements flowing randomly from one part of the body to another (28). The HD patients recruited in this study were all chorea-dominant rather than “juvenile HD” or “Westphal variant” that present with an akinetic rigid syndrome usually before the age of 20 (29). All dystonia patients in this study had primary cervical dystonia characterized by predominance of tonic components over phasic components. Therefore, the differences in LFP patterns between these groups likely reflect these distinct motor syndromes.

### Low frequency activity

This study implicates GPi low frequency activity in dystonia (defined as 6–14 Hz although there are no clear boundaries within this frequency band). Our results address two important questions regarding neural oscillations in hyperkinetic diseases. The first question is whether elevated low frequency power is disease-specific, syndrome-specific, or merely a reflection of current movement status. Several previous studies reported that low frequency oscillations and EMG synchronization in GPi are correlated during involuntary dystonic muscle contractions, suggesting that the low frequency band contributes to the physiopathology of dystonia (30, 31). In contrast, another study found that alpha band power is elevated in HD (21). In our study, however, alpha band power was lower in HD than dystonia. This discrepancy may be related to the difference in HD-associated dystonia between studies. The HD patients recruited in this study had less severe dystonic symptoms compared to the dystonia patients, so the elevated low frequency band power may reflect the dystonic syndrome rather than the disease itself. The second question is whether idiopathic dystonia responds well to GPi-DBS (32–34), as the dystonic syndrome in HD does not (35). Based on this study, we conclude that the dystonic syndrome in HD is not that prominent compared to dystonia and DBS may most strongly suppress the prominent frequency band. The total power at 6–13 Hz was significantly lower in HD patients than dystonia patients, so the dystonia syndrome in HD may not respond as well as chorea to DBS. The hypothesis requires further study for verification. The low-frequency band LZC was also lower in dystonia than HD, indicating a more regular pattern. A previous study found that the theta band significantly correlated with the severity of dystonic syndrome in cervical dystonia patients (10). In our study, no significant correlation was found between the TWSTRS and 6–14 Hz power or the 6–14 Hz/26–43 Hz power ratio, which might due to the relative small number of patients. The other possibility is that the dystonia patients recruited in this study were of the primary cervical dystonia type, in which tonic components are predominant over phasic components.

### Beta activity

Beta activity is strongly implicated in movement inhibition (particularly beta activity in motor cortex and within STN). Indeed, beta activity increases during movement antagonism and termination (36). In addition, Pogosyan et al. demonstrated that enhanced synchronization of beta oscillation induced by transcranial electric stimulation slows movement (37). Moreover, beta is thought to decrease information relay via top-down communication. It was suggested that beta oscillations inhibit processing and are reduced focally to allow optimal information relay (38). Collectively, these findings indicate that beta activity is more a normal physiomarker used to sustain normal action and is desynchronized when a new action program is activated. Alternatively, hyper-elevated beta power damages the normal network and results in voluntary action inhibition.

Recent studies have found subtle differences in cortical topography, and estimates of time delays underlying STN cortical beta coherence hint at a contribution of the indirect pathway to low-beta oscillations (13–25 Hz) and the hyper-direct pathway for high-beta oscillations (26–35 Hz) (39). Severity of bradykinesia and rigidity appear closely linked to enhanced activity, specifically in the low-beta range (40, 41), whereas high-beta has been linked to FOG (42). The beta band can be subdivided into sub-bands and the low-beta band exhibits desynchronization in fast-movement tasks (43, 44). Early desynchronization in low-beta occurs when more challenging, complex, and demanding tasks must be performed (45, 46). In this study, HD patients showed no differences in low-beta frequency band (15–25 Hz) power and LZC, which may reflect the early disease stage of our HD patient group. Mounting experimental evidence supports the notion that indirect striatal projection neurons (iSPNs) and direct striatal projection neurons (dSPNs) help to respectively suppress and promote cortical selection of particular actions (6, 47). In the early stages of HD, chorea (hyperkinesia) is one of the most prominent symptoms. This feature is strongly correlated with iSPN dysfunction (48), which is impaired earlier as revealed by the course of neuropathology in HD. The severities of bradykinesia and rigidity syndrome were similar between the HD and dystonia group in our study, which may account for the lack of difference in low-beta band power and low-beta band LZC between groups. Our study also implies that the hyper-direct pathway may be involved in the pathogenesis of HD, which is correlated with greater high-beta band power and lower high-beta band LZC (26–35 Hz) than dystonia. A regular high-beta band pattern is more prominent in HD patients. Hyper-direct pathway drive is supposed to be a necessary requirement for developing exaggerated beta activity in the STN (49). The elevated high-beta band oscillation may disrupt normal information transfer, and this disruption is more severe in HD than dystonia. This high-beta band activity together with the elevated high-gamma band oscillation may lead to chorea. So, there is no posture to alleviate chorea, unlike the dystonic syndrome (50). Moreover, the 26–43 Hz power was positively correlated with the UHDRS chorea subscore while the 6–14 Hz/26–43 Hz power ratio was negatively correlated with the UHDRS chorea sub-score.

### Gamma activity

Gamma oscillation synchronizes prior to movement onset and is believed to promote movement (51). Power increases of subthalamic gamma oscillations are associated with increments of movement performance and enhanced reaction time in response to intense auditory stimuli (52). The gamma activity is lateralized to the contralateral hemisphere during active motion (53), suggesting promotion of normal voluntary movement.

LFP recordings from cortex and basal ganglia in a rodent model of parkinsonism revealed that levodopa-induced dyskinesia is associated with rising gamma band energy. Dyskinesia syndrome resembles chorea syndrome in that both are characterized by involuntary choreiform movements (54). We found a strong correlation between low-gamma and chorea in HD. On the other hand, one study comparing the resting state with a normal/slow and fast horizontal line drawing movement found suppressed low-beta and increased high-beta and gamma band activity on the contralateral side while the movement speed was increasing. The low-beta band desynchronization and gamma band synchronization are associated with execution and scaling of motor tasks (55, 56).

Collectively, these studies indicate that gamma activity is prokinetic. In our study, the 35–43 Hz power was higher in HD than dystonia, while LZC was lower. A previous study of GPI-LFPs in a HD patient also found increased power in the 35–45 Hz band (21). The scaling of chorea is larger than dystonia. We speculate that the chorea syndrome of HD is more hyperactive due to the elevated gamma activity.

### The potential biomarker for different syndrome

Continuous DBS can induce side effects like psychiatric symptoms and dysarthria, suggesting that adaptive DBS may be more effective (57, 58). Indeed, aDBS has already shown greater clinical efficacy for advanced PD than cDBS (59), likely because aDBS more effectively disrupts long beta-oscillations (60). The LFP identification is also very important for adaptive DBS. However, whether the oscillatory activities in the same site are different in the two types of hyperkinetic diseases or not remains unclear. And if they are, what are the relationships between the characteristic oscillatory activities and the clinical representation of hyperkinetic symptoms? Our study showed a relative clear boundary between chorea and dystonic syndrome. The ROC curve also implied that the 6–14 to 26–43 Hz power ratio can distinguish these two syndromes. The high beta and low gamma frequency band in resting state might be a biomarker of chorea. We can use this biomarker to detect the chorea severity variation. Thus, these LFP-derived measures may have potential utility as biomarkers to identify syndromes of hyperkinetic diseases.

## Limitation

There are some confounding factors that cannot be ignored. First, data on dystonia and HD patients were acquired in different countries, suggesting a possible influence of ethnic differences. Second, the channels we selected did not match the anatomic location accurately due to the thick acquisition slices. Spectral normalization and LZC help address this issue to some extent as LZC analysis is independent of infinite amplitude. Another limitation is that we did not analyze the influence of DBS on LFP in these two diseases, which is planned for future studies.

## Conclusion

In conclusion, our data imply that the spectral patterns in GPi differ between chorea-dominant HD and primary cervical dystonia. Chorea is more likely related to elevated high-beta and gamma band activities while the dystonic syndrome is more likely related to elevated low frequency band activity. The specific characteristics of LFP oscillations appear more closely related to the syndrome than the specific disease. These LFP measures advance our understanding of the abnormalities in basal ganglia signaling that underlie HD symptoms and could be useful biomarkers for setting adaptive DBS parameters.

## Author contributions

GZ designed and conducted the experiment, collected MRI and clinical information, and drafted the manuscript. XG collected electrophysiological data, analyzed and interpreted the data. ZT analyzed and interpreted the data. YC, RZ and XW collected the data. TA performed the DBS and clinical evaluation. SW supervised the design of experiment and data analysis, and revised the manuscript. JZ supervised the design of experiments, performed the DBS and clinical evaluation, and revised the manuscript.

### Conflict of interest statement

The authors declare that the research was conducted in the absence of any commercial or financial relationships that could be construed as a potential conflict of interest.
